# Matrine Directly Activates Extracellular Heat Shock Protein 90, Resulting in Axonal Growth and Functional Recovery in Spinal Cord Injured-Mice

**DOI:** 10.3389/fphar.2018.00446

**Published:** 2018-05-07

**Authors:** Norio Tanabe, Tomoharu Kuboyama, Chihiro Tohda

**Affiliations:** Division of Neuromedical Science, Department of Bioscience, Institute of Natural Medicine, University of Toyama, Toyama, Japan

**Keywords:** matrine, spinal cord injury, axonal growth, chondroitin sulfate proteoglycan, DARTS, heat shock protein 90, HSP90 activator, extracellular HSP90

## Abstract

After spinal cord injury (SCI), reconstruction of neuronal tracts is very difficult because an inhibitory scar is formed at the lesion site, in which several axonal growth inhibitors, such as chondroitin sulfate proteoglycans (CSPG), accumulate. We previously found that matrine, a major alkaloid in *Sophora flavescens*, enhanced axonal growth in neurons seeded on CSPG coating. The aims of this study were to investigate therapeutic effects of matrine in SCI mice and to clarify the underlying mechanism. Matrine was orally administered to contusion SCI mice. In the matrine-treated mice, motor dysfunction of the hindlimbs was improved, and the density of 5-HT-positive tracts was increased in the injured spinal cord. We explored putative direct binding proteins of matrine in cultured neurons using drug affinity responsive target stability (DARTS). As a result, heat shock protein 90 (HSP90) was identified, and matrine enhanced HSP90 chaperon activity. We then presumed that extracellular HSP90 is a matrine-targeting signaling molecule, and found that specific blocking of extracellular HSP90 by a neutralizing antibody completely diminished matrine-induced axonal growth and SCI amelioration. Our results suggest that matrine enhances axonal growth and functional recovery in SCI mice by direct activation of extracellular HSP90. Matrine could be a significant candidate for therapeutic drugs for SCI with a novel mechanism.

## Introduction

Spinal cord injury (SCI) is accompanied by refractory deficits of motor, sensory, and autonomic functions. These dysfunctions are caused by disruptions of descending and/or ascending tracts. Reconstruction of the neuronal tracts is very difficult because an inhibitory environment, called a glial scar, is formed at the lesion site. The scar tissue contains several axonal growth inhibitors, such as chondroitin sulfate proteoglycan (CSPG) (Dyck and Karimi-Abdolrezaee, 2015). Currently, there are no drugs that are recommended in clinical guidelines as functional ameliorants for SCI (Walters et al., 2013). The development of novel clinical drugs effective for functional recovery in SCI is therefore required and eagerly expected.

In a previous study, we found that the water extract of dried roots of *Sophora flavescens*, a traditional medicine used in China, Korea, and Japan, enhanced axonal growth of cultured cortical neurons on a CSPG coating (Tanabe et al., 2016). Administration of *S. flavescens* extract to SCI mice increased axonal density [5-hydroxytryptamine (5-HT)-positive tracts] at the lesion site and improved the motor dysfunction. Furthermore, matrine, a major constituent in *S. flavescens*, also showed axonal growth activity *in vitro*. However, the effects of matrine in SCI mice have not been investigated, and the mechanism behind matrine-induced axonal growth is still unknown.

Matrine is a small compound (molecular weight: 248) classified as a lupin alkaloid that shows various pharmacological effects, including anti-cancer (Ma et al., 2008; Liu et al., 2010; Liang et al., 2012), anti-inflammatory (Zhang et al., 2011; Sun et al., 2016), and anti-virus (Yang et al., 2012) effects. In recent years, several basic studies showed direct binding proteins of matrine. Epidermal growth factor receptor (Wang et al., 2010), vasodilator-stimulated phosphoprotein (Zhang et al., 2013), and annexin A2 (Wang et al., 2017) were identified as the direct binding proteins of matrine in cancer cells, and matrine inhibited these protein-induced cell events. Cui et al showed that matrine bound to amyloid β peptide and receptor for advanced glycation end products, and the cell signaling stimulated by these proteins were inhibited by matrine in neuroblastoma cells (Cui et al., 2017). Furthermore, the activity of trypsin was directory suppressed by matrine treatment (Hu et al., 2015), and a computational docking simulation indicated a high affinity of matrine to heat shock protein 90 (HSP90) (Zeng et al., 2015). Although it is important to identify the direct target molecule linking to each pharmacological effect of matrine in order to understand its mechanistic diversity, nobody has investigated the direct binding proteins of matrine in primary cortical neurons, and it is unclear what proteins mediate matrine-induced axonal growth.

In this study, we aimed to investigate the effect of matrine in SCI model mice, and identify the direct target protein contributing to the pharmacological effects.

## Materials and methods

All experiments were performed in accordance with the Guidelines for the Care and Use of Laboratory Animals of the Sugitani Campus of the University of Toyama. All protocols were approved by the Committee for Animal Care and Use of the Sugitani Campus of the University of Toyama; the approval numbers for the animal experiments are A2013INM-1 and A2016INM-3. All efforts were made to minimize the number of animals used.

### SCI experiments

**Figure 1:**
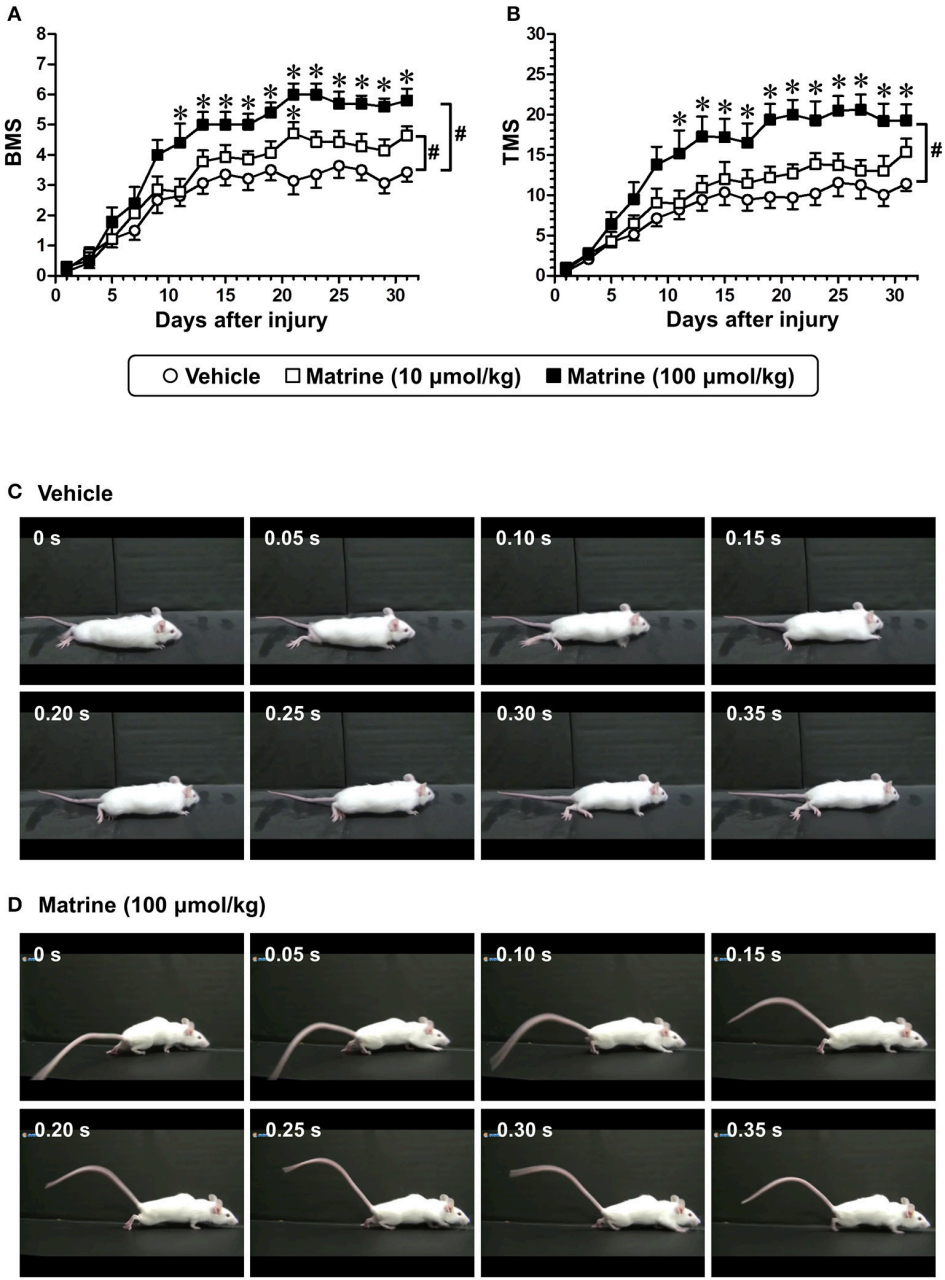
Matrine enhances recovery of motor function of hindlimbs in spinal cord-injured (SCI) mice. Matrine (10 or 100 μmol kg^−1^ day^−1^) or vehicle solution (saline) was constitutively administered in oral form 1 h after the injury for 31 days. The Basso Mouse Scale (BMS, **A**) and the Toyama Mouse Score (TMS, **B**) were measured to evaluate motor functions of the hindlimbs. # *p* < 0.05, vs. vehicle, drug × day interaction analyzed by two-way repeated measures ANOVA. ^*^p < 0.05, vs. vehicle, *post hoc* Bonferroni test. Vehicle group: white circles, 7 mice, 14 hindlimbs, *n* = 14. Matrine (10 μmol kg^−1^) group: white squares, 7 mice, 14 hindlimbs, *n* = 14. Matrine (100 μmol kg^−1^) group: black squares, 5 mice, 10 hindlimbs, *n* = 10. The movement of a representative SCI mouse treated with vehicle **(C)** or matrine (100 μmol kg^−1^) **(D)** was captured at 31 days after injury. The images are sequential for 0.35 s.

Contusive SCI mice by a weight drop, a major model of SCI (Zhang et al., 2014), were chosen. Eight-week-old female ddY mice (28–33 g) were purchased from Japan SLC (Shizuoka, Japan). The mice were housed in a constant environment (22 ± 2°C, 50 ± 5% humidity, 12-h light cycle starting at 07:00) with free access to food and water. Under anesthetization with trichloroacetaldehyde monohydrate, the mice were laminectomized and fixed on a stereotaxic instrument (Narishige, Tokyo, Japan). Exposed spinal cord at the T10-11 level was contused by dropping a rod (the tip diameter; 1 mm), through a vertical cylinder. Less severe contusion (Figure 1) was produced by a drop with a rod of 6.5 g from a 2-cm height, and severe contusion (**Figure 6**, Supplementary Figure 5) was produced by a drop of 13.6 g from a 3-cm height. During and after surgery, the mice were kept on a hotplate to maintain body temperature. Before drug treatment, the mice were randomly assigned to the different treatment groups. Matrine (10 and 100 μmol kg^−1^ day^−1^; Tokyo Chemical Industry, Tokyo, Japan) or vehicle solution (saline) was orally and continually administered to SCI mice from 1 h after the injury to the end of the experiment, once a day. The number of mice for each treatment group was indicated in the figure legends. Group sizes were equal by design (Figure 1: 8 mice/ a group, **Figure 6**: 7 mice/ a group), and any variations are due to experimental losses. The motor function of the hindlimbs was assessed using the Basso Mouse Scale (BMS) (Basso et al., 2006) and the Toyama Mouse Score (TMS) (Shigyo et al., 2014) in an open field (black color, 50.0 × 42.5 × 15.0 cm) under 500-lux illumination.

### Immunohistological analysis

Thirty-one days after the SCI, the mice were anesthetized with trichloroacetaldehyde monohydrate, and were transcardially perfused with ice-cold saline and 4% paraformaldehyde (PFA). Spinal columns were isolated and kept in 4% PFA at 4°C. Spinal cord tissues around the lesion were separated and fixed further with 4% PFA overnight. The spinal cords were immersed in 10, 20, and 30% sucrose-phosphate buffered saline (PBS) successively at 4°C and embedded in Tissue-Tek Optimal Cutting Temperature Compound (Sakura Finetek Japan, Tokyo, Japan). The spinal cords were cut into 14-μm successive sagittal sections using a CM3050S cryostat (Leica, Heidelberg, Germany). The slices were post-fixed with 4% PFA for 60 min at room temperature and immunostained with a rabbit anti-5-hydroxytryptamine (5-HT, a marker of the raphespinal tract) polyclonal antibody (dilution 1:500; Immuno Star, Hudson, WI, USA), a mouse anti-glial fibrillary acidic protein (GFAP, a marker of reactive astrocytes), monoclonal antibody (clone G-A-5; dilution 1:1000; Sigma-Aldrich, St. Louis, MO, USA), and a mouse anti-CSPG monoclonal antibody (clone CS-56; dilution 1:500; Sigma-Aldrich). As secondary antibodies, Alexa Fluor 488-conjugated goat anti-rabbit IgG (dilution 1:400; Thermofisher Scientific, Waltham, MA, USA), Alexa Fluor 594-conjugated goat anti-mouse IgG_1_ (dilution 1:400; Thermofisher Scientific), and Alexa Fluor 350-conjugated goat anti-mouse IgM (dilution 1:400; Thermofisher Scientific) were used. Fluorescence images were captured using an inverted microscope (Axio Observer Z1; Carl Zeiss, Oberkochen, Germany), a 20 × NA 0.8 objective lens (Plan-Apochromat; Carl Zeiss), and a charge-coupled device camera (AxioCam MRm; Carl Zeiss). Successive z-stack images around the lesion site were obtained, overlaid, and tiled using Axio Vision 4.8 software (Carl Zeiss). A glial scar was defined as the area surrounded by GFAP-positive reactive astrocytes. The size of the glial scar and the expression level of CSPG in the glial scar were measured using ImageJ software (NIH, Rockville, MD, USA). The area of 5-HT-positive fibers in the glial scar and in the area 1.5–2.0 mm caudal from the lesion center was also quantitated using ImageJ software.

### Primary culture of cortical neurons

Culture dishes were coated with Hank's buffered salt solution (HBSS; Thermofisher Scientific) containing 5 μg ml^−1^ poly-_D_-lysine (PDL; Sigma-Aldrich) and 2.0 μg ml^−1^ aggrecan (Sigma-Aldrich), one of the CSPGs, overnight at 37°C. Embryos of ddY mice (Japan SLC) were obtained 14 days after gestation. Cortices without dura mater were isolated, minced, dispersed, and cultured on the dishes with neurobasal medium (Thermofisher Scientific) containing 12% horse serum (Thermofisher Scientific), 2 mM _L_-glutamine, and 0.6% _D_-glucose at 37°C in a humidified incubator at 10% CO_2_. Five hours after the seeding, the medium was replaced with fresh neurobasal medium containing 2% B-27 supplement (Thermofisher Scientific) instead of horse serum. The purity of neurons was 75% on PDL coating and 57% on CSPG coating at 7 days after seeding (Supplementary Figure 1).

### Drug affinity responsive target stability (DARTS) analysis

A DARTS analysis was performed as previously described (Lomenick et al., 2009), with slight modifications.

Cortical neurons were cultured on 10-cm culture dishes (Falcon, Franklin Lakes, NJ, USA) coated with PDL and CSPG, as described above. Three days after seeding, the neurons were treated with matrine (100 μM) or vehicle solution (0.1% DMSO) for 30 min. After washing with PBS, the neurons were lysed with M-PER (Thermofisher Scientific) containing a protease and phosphatase inhibitor cocktail (Thermofisher Scientific). Protein concentration in the lysates was measured using Pierce 660 nm Protein Assay Reagent (Thermofisher Scientific). The lysates were mixed with thermolysin (Wako, Osaka, Japan), which was dissolved in reaction buffer [50 mM Tris-HCl (pH 8.0), 50 mM NaCl, 10 mM CaCl_2_], at a ratio of 1 mg to 92 pU. The mixture was incubated for 30 min at 37°C. To stop the proteolysis, 0.5 M ethylenediaminetetraacetic acid (EDTA) (pH 8.0) was added to the mixture at a 1:10 ratio. The samples were analyzed by sodium dodecyl sulfate-polyacrylamide gel electrophoresis (SDS-PAGE). The separated proteins were stained using a silver staining kit (SilverQuest; Thermofisher Scientific) to compare the thickness of vehicle-treated to that of matrine-treated lysates. A protein band which was thinner in the matrine-treated lysate than in the vehicle-treated lysate was cut out. Nano-liquid chromatography-tandem mass spectrometry (Nano-LC-MS/MS) analysis of the band was performed by Japan Bio Services (Asaka, Japan). The band underwent decoloration, deoxidization of cysteine residues, alkylation, and in-gel digestion by trypsin. The digested peptides were extracted from the gel and desalted. After that, the samples were analyzed using nano-LC-MS/MS with Mascot search (MS/MS Ion Search).

After proteolysis, the lysates treated with matrine (1, 10, and 100 μM), geldanamycin (1 μM) or vehicle solution were analyzed by SDS-PAGE followed by western blot for HSP90. The lysates were separated by SDS-PAGE and transferred to a nitrocellulose membrane (Bio-Rad, Hercules, CA, USA). The membrane was blocked with 5% skim milk in Tris-buffered saline containing 0.1% Tween 20 at room temperature (~20–24°C) for 1 h, and was reacted with a mouse anti-HSP90 α/β monoclonal antibody (clone F-8; dilution 1:500; Santa Cruz, Dallas, TX, USA) in Can Get Signal Solution 1 (Toyobo, Osaka, Japan) at 4°C overnight. As a secondary antibody, a horseradish peroxidase-conjugated goat anti-mouse IgG (dilution 1:2000; Santa Cruz) was used in Can Get Signal Solution 2 (Toyobo). Specific bands were visualized using the Enhanced Chemiluminescence Prime Western Blotting Detection System (GE-Healthcare, Pittsburgh, PA, USA) and captured with LAS4000 (GE Healthcare). Signal intensities were quantified using a CS analyser (ATTO, Tokyo, Japan).

### Luciferase refolding assay

The luciferase refolding assay was performed as previously described (Eachkoti et al., 2014) with slight modifications.

Firefly luciferase (Sigma-Aldrich; cat#L9506) at a concentration of 0.5 mg ml^−1^ was dissolved in stability buffer (25 mM Tricine-HCl [pH 7.8], 8 mM MgSO_4_, 0.1 mM EDTA, 10 mg ml^−1^ bovine serum albumin (BSA) [Wako; cat#9048-46-8]). After the luciferase was completely dissolved, 10% glycerol and 1% triton X-100 were added. The luciferase mix was aliquoted, frozen in liquid nitrogen and stored at −80°C.

The luciferase mix (0.1 ml) was heated to 41°C for 3 min to denature the luciferase mildly, and was then mixed with a mixture of 100 mM Tris-HCl (pH 7.7), 10 nM Mg(OAc)_2_, 375 mM KCl, 15 mM ATP, and 25 mM creatine phosphate (6.4 ml), 2.5 mg ml^−1^ creatine phosphokinase (0.64 ml, dissolved in 50% glycerol; Sigma-Aldrich), and ultrapure water (0.86 ml). The denatured luciferase mix was aliquoted, frozen in liquid nitrogen, and stored at 80°C.

Immediately before use, a rabbit reticulocyte lysate (RRL; Promega, Madison, WI, USA; cat#L4960) was diluted with 20 mM Tris-HCl (pH 7.4) containing 75 mM KCl at a ratio of 1 to 3. Ten microliters of the RRL or TBS/HbBSA (20 mM Tris-HCl [pH 7.5], 150 mM NaCl, 1% hemoglobin [Sigma-Aldrich; cat#H2625] and 4% BSA) was mixed with 20 μl of matrine (100, 500, and 1,000 μM), geldanamycin (1, 10, and 100 μM; Tokyo Chemical Industry) or vehicle solution [f.c. 1% dimethyl sulfoxide (DMSO) in ultrapure water] in a 96-well white plate (Corning, Corning, NY, USA). The plate was incubated at 37°C for 30 min. Ten microliters of the denatured luciferase mix was added to each well. After incubation at room temperature for 30 min, luciferase activity was measured by adding 40 μl of a detection buffer (Luciferase Assay Reagent included in Luciferase Assay System; Promega; cat#E1500). Light emission intensity was detected using a multi-mode micro plate reader (Filter Max F5; Molecular Devices, Sunnyvale, CA, USA). To estimate the amount of luminescence produced by refolded luciferase, the difference of light emission intensity between RRL-containing wells and TBS/HbBSA-containing (negative control) wells was calculated.

### CSPG coating assay

Cortical neurons were cultured for 3 days on 8-well chamber slides (Falcon) coated with PDL and CSPG, as described above. A mouse anti-HSP90α/β antibody (1 ng ml^−1^) or a normal mouse IgG (1 ng ml^−1^; Santa Cruz) was applied to the cells. Thirty minutes later, matrine (10 μM) or vehicle solution (0.1% DMSO) was added. After 4 additional days of incubation, the cells were fixed with 4% PFA and immunostained with a mouse anti-phosphorylated neurofilament H (pNF-H, an axonal marker) monoclonal antibody (dilution 1:250; clone SMI-35; BioLegend, San Diego, CA, USA), and a rabbit anti-microtubule-associated protein 2 (MAP2, a neuronal marker) polyclonal antibody (dilution 1:2000; Abcam, Cambridge, UK) at 4°C overnight. Alexa Fluor 488-conjugated goat anti-mouse IgG (dilution 1:400; Thermofisher Scientific) and Alexa Fluor 594-conjugated goat anti-rabbit IgG (dilution 1:400; Thermofisher Scientific) were used as secondary antibodies. Counterstaining with 4′, 6-diamidino-2-phenylindole (DAPI, a nuclei marker; 0.1 μg ml^−1^; Enzo Life Science, Farmingdale, NY, USA) was also performed. Fluorescence images were captured using an inverted microscope (Axio Observer Z1), a 10 × NA 0.45 objective lens (Plan-Apochromat; Carl Zeiss), and a charge-coupled device camera (AxioCam MRm). On each image, the total axon length was automatically measured using the MetaMorph analyser (Molecular Devices), and the number of neurons was manually counted to calculate the axonal density per neuron.

### Continuous I.C.V. infusion

A mini-osmotic pump (Alzet, Cupertino, CA, USA; cat#1004) was connected to a cannula (Brain Infusion Kit 3; Alzet) via a 5-cm piece of polyvinylchloride tube (Alzet). The pumps were filled with 164 ng ml^−1^ mouse anti-HSP90α/β monoclonal antibody, vehicle solution (artificial cerebrospinal fluid (aCSF) [148.3 mM NaCl, 3 mM KCl, 1.4 mM CaCl_2_, 0.8 mM MgCl_2_, 0.75 mM Na_2_HPO_4_, and 0.195 mM NaH_2_PO_4_]) or normal mouse IgG diluted in aCSF. The production rate of CSF in the mouse brain is 18 μl h^−1^. The infusion rate of the mini-osmotic pump was 0.11 μl h^−1^. Because the anti-HSP90 antibody was diluted by CSF at a ratio of 1:164, the concentration of anti-HSP90 antibody in CSF was 1 ng ml^−1^, which is the effective dose of the antibody for neutralization *in vitro* (**Figure 5**). The filled pumps were incubated in sterile saline at 37°C for more than 48 h.

Before the SCI surgery, ddY mice were placed on a stereotaxic instrument to keep the head in a fixed position. The scalp was shaved and cut to expose the skull. To detain the cannula into the right lateral ventricle (anteroposterior: −0.22 mm, mediolateral: +1 mm, dorsoventricular:−2.5 mm), a burr hole was drilled into the skull using a dental drill. After the SCI surgery, the mice were randomly assigned to the different treatment groups. The cannula was positioned and fixed on the skull using Aron Alpha A “Sankyo” adhesive (Daiichi Sankyo, Tokyo, Japan). The pump was implanted under the dorsal skin of the back of the mouse. During and after surgery, mice were placed on a heating pad to maintain body temperature. When the cannulas were broken until the end of experiment, the mice were excluded from statistical analysis.

### Statistical analysis

Statistical comparisons were performed using two-way repeated measures analysis of variance (ANOVA) with *post hoc* Bonferroni tests, unpaired two-tailed *t*-test, paired two-tailed *t*-test or a one-way ANOVA with *post hoc* Dunnett's tests using GraphPad Prism 5 (GraphPad Software, San Diego, CA, USA). A *p* < 0.05 was considered significant. The data are presented as the mean ± standard error of the mean (SE).

## Results

### Matrine increases the density of 5-HT-positive raphespinal tracts and enhances functional recovery in SCI mice

To investigate the effect of matrine on SCI mice, matrine (10 and 100 μmol kg^−1^ day^−1^) or vehicle solution (saline) was orally administered for 31 days following injury (less severe condition). The motor functions of both hindlimbs were evaluated independently using the BMS and the TMS. Compared with vehicle treatment, high-dose matrine treatment led to a significant improvement in motor functions as measured by the BMS (Figure 1A) and the TMS (Figure 1B), while low-dose matrine treatment led to a slightly increased BMS score (Figure 1A). The two-way repeated measures ANOVA yielded the following F and *p* values for the drug × day interaction between the vehicle and each matrine treatment: [vs. 10 μmol kg^−1^ matrine] *F*_(15, 390)_ = 2.076, *p* = 0.0104 in BMS; *F*_(15, 390)_ = 1.108, *p* = 0.3467 in TMS; [vs. 100 μmol kg^−1^ matrine] *F*_(15, 330)_ = 6.427, *p* < 0.0001 in BMS; *F*_(15, 330)_ = 6.132, *p* < 0.0001 in TMS. At 31 days after the injury, vehicle-administered mice were not able to lift their trunk when walking (Figure 1C; Supplementary Video 1). In contrast, mice that had been administered a high dose of matrine walked while supporting their trunk (Figure 1D; Supplementary Video 2).

**Figure 2:**
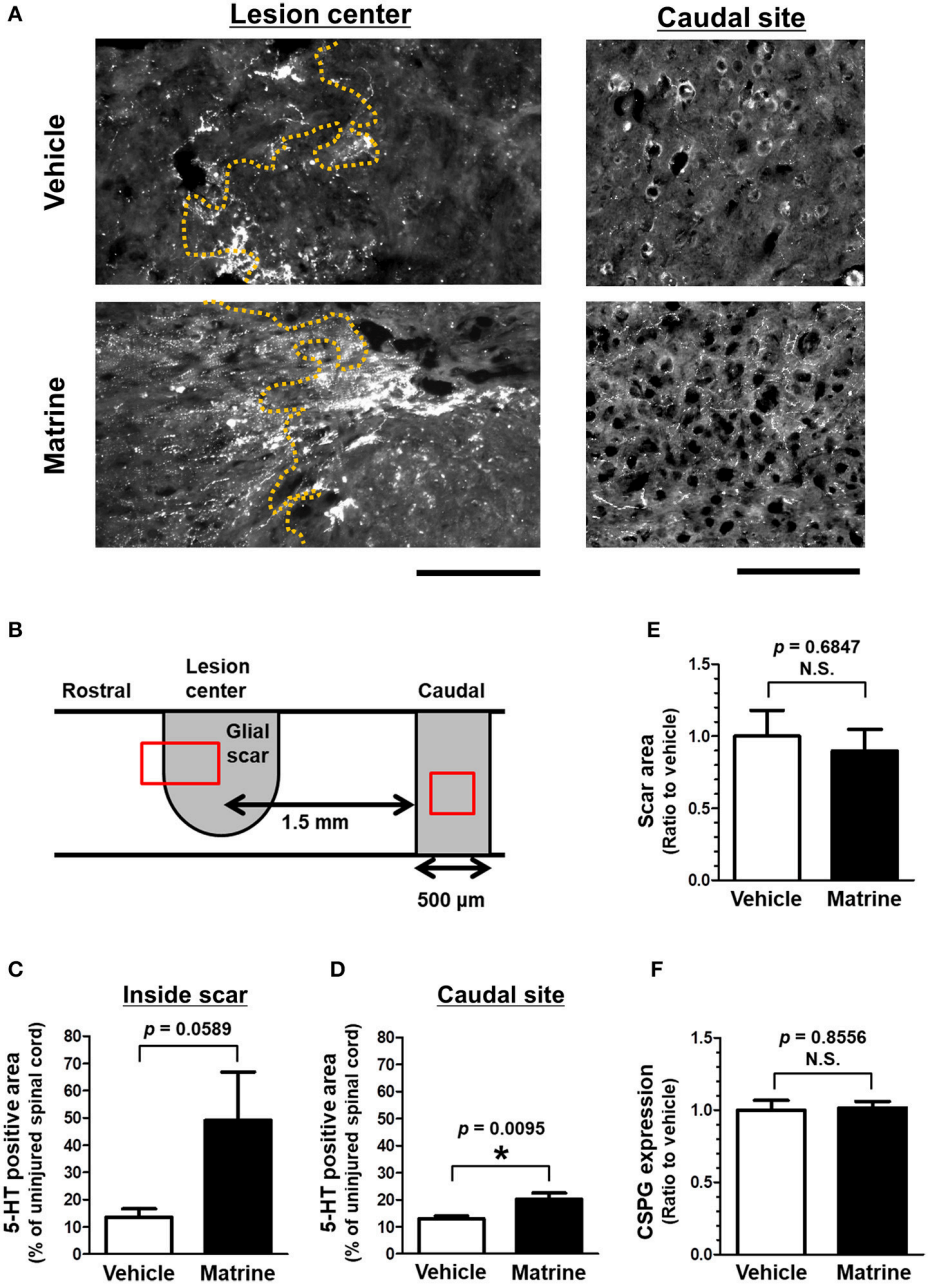
Matrine increases 5-HT-positive raphespinal tracts in SCI mice. At 31 days after injury, injured spinal cords were isolated from SCI mice that were treated with vehicle or matrine (100 μmol kg^−1^). Sagittal sections of the spinal cords were immunostained for 5-HT, glial fibrillary acidic protein (GFAP), and chondroitin sulfate proteoglycan (CSPG). **(A)** Representative images of immunohistochemistry for 5-HT are shown. The yellow dotted lines are borderlines of the glial scar. The left direction of the images is rostral, and the upper side is dorsal. Scale bars = 200 μm. **(B)** The glial scar was defined as the inside space surrounded by GFAP-positive astrocytes, and the caudal site was defined as the area 1.5–2.0 mm away from the lesion center. The red squares indicate the areas shown in **(A)**. **(C,D)** The area of 5-HT-positive fiber-like stains was quantified inside the glial scar and at the caudal site. **(E)** The size of the glial scar was measured. **(F)** The level of CSPG expression at the glial scar was measured. ^*^p < 0.05, unpaired *t*-test (two-tailed). *n* = 6 (vehicle) or 5 (matrine).

Thirty-one days after the injury, the injured spinal cords were isolated, and sagittal sections were analyzed by immunohistochemistry. To evaluate the outgrowth of descending tracts, 5-HT-positive raphespinal tracts were visualized (Figure 2A, Supplementary Figure 2). Raphespinal tracts are important for the regulation of locomotor function (Liu and Jordan, 2005). The area of 5-HT-positive fiber-like stains was measured in the glial scar, which was defined as the area surrounded by GFAP-positive reactive astrocytes, and within the area 1.5–2.0 μm caudal from the lesion center (Figure 2D). Matrine administration increased the area of 5HT-positive and fiber-like stains inside the glial scar (Figure 2B), and also within the caudal area further away from the lesion center (Figure 2C), compared to vehicle treatment. The size of the glial scar did not differ significantly between the groups (Figure 2E). In addition, matrine treatment had no effect on CSPG expression levels at the lesion site (Figure 2F). These results suggest that matrine increases the density of raphespinal tracts in the injured spinal cord, which might lead to an improvement in motor functions in SCI mice.

### Matrine interacts with HSP90 and enhances its chaperon activity

**Figure 3:**
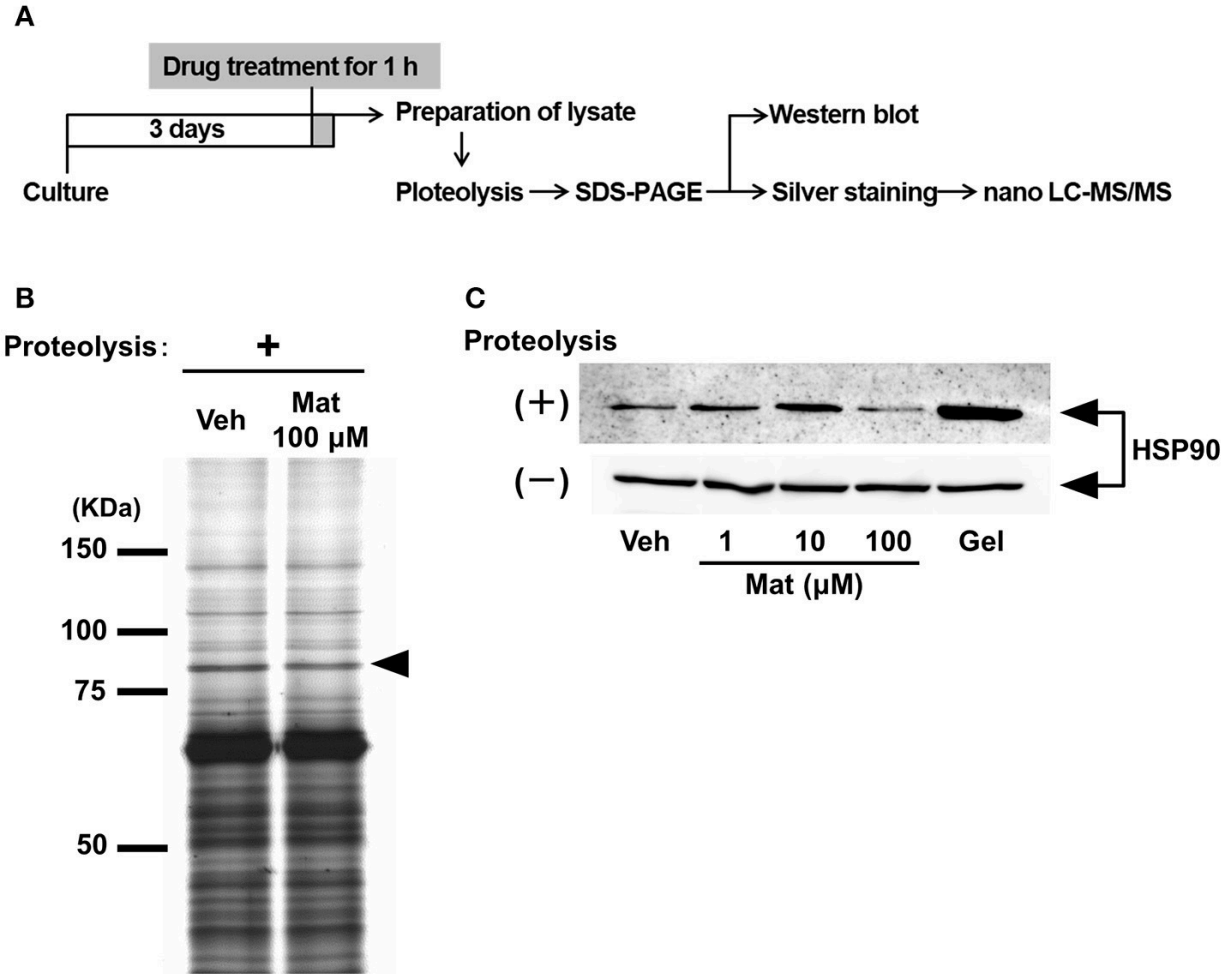
Matrine binds to heat shock protein (HSP) 90 in neurons. **(A)** Scheme of the drug affinity responsive target stability (DARTS) experiment. Mouse cortical neurons were cultured on CSPG-coated dishes for 3 days. Thirty minutes after the treatment with matrine (Mat; 1, 10, and 100 μM), geldanamycin (1 μM), or vehicle (Veh), the neurons were lysed to prepare cell lysate. The lysates were proteolysed with thermolysin and ran on an SDS-PAGE gel, and protein patterns were visualized by silver staining. Bands whose thickness was different between vehicle- and matrine-treated lysate were identified on the gel and were analyzed by nano-LC-MS/MS. Vulnerability of candidate proteins against proteolysis was confirmed by DARTS reaction and followed western blotting. **(B)** A representative image of an SDS-PAGE gel stained by silver staining is shown. The black arrowhead indicates the bands analyzed by nano-LC-MS/MS. **(C)** HSP90 was detected by western blot in the lysates with or without proteolysis. Twenty microgram of digested lysates after proteolysis and 4 μg of naive lysates were loaded.

To clarify the molecular mechanism of matrine-induced axonal growth, we explored an initial target molecule of matrine in neurons using DARTS, which is a method to identify comprehensively putative direct binding proteins of a small molecule compound. Since the binding of a compound to a protein possibly changes the conformation of the protein and its proteolytic sensitivity, the bound proteins become resistant or vulnerable against proteolysis. We prepared lysates from primary cultured cortical neurons treated with matrine or vehicle solution. After proteolysis by thermolysin, a thinner band was detected at 75–100 kDa molecular weight in the matrine-treated lysate than in the vehicle-treated lysate (Figure 3B). Nano-LC-MS/MS analysis indicated that this band is HSP90. To confirm the result of the nano-LC-MS/MS analysis, western blot for HSP90 was performed after the DARTS reaction. Multi doses of matrine (1, 10, and 100 μM) and geldanamycin (1 μM), a HSP90 inhibitor, were investigated for estimating the stability of HSP90 in DARTS (Figure 3C). Although HSP90 was destabilized by treatment with 100 μM matrine, 1, and 10 μM doses of matrine stabilized HSP90. Geldanamycin also stabilized HSP90. The destabilized HSP90 by 100 μM matrine was confirmed by six experiments (Supplementary Figure 3). The data showed that 100 μM matrine significantly destabilized HSP90 against proteolysis. These results suggest that matrine might bind to HSP90 directly.

**Figure 4:**
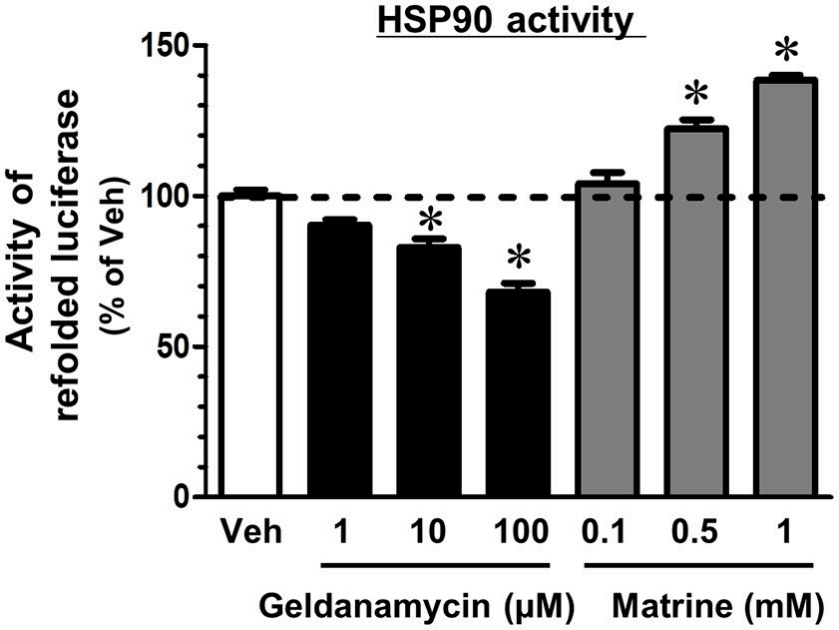
Matrine activates the HSP90 folding function. A luciferase refolding assay was performed to assess the effect of matrine on HSP90 folding activity. Rabbit reticulocyte lysate, which was pre-treated with matrine (0.1, 0.5, and 1 mM), geldanamycin (1, 10, and 100 μM), or vehicle solution was incubated with thermally denatured luciferase for 30 min. Luciferase activity was then measured using the luciferase assay kit. The luminescence value of denatured luciferase without the refolding reaction was defined as 0%. ^*^p < 0.05, vs. vehicle, one-way ANOVA followed by *post hoc* Dunnett's test. *n* = 5.

HSP90 is a molecular chaperon that folds many kinds of proteins (Taipale et al., 2010). Another group reported that a computational docking simulation indicated a high affinity of matrine to HSP90 (Zeng et al., 2015), which supports our result in the DARTS experiment; however, the effect of matrine on HSP90 chaperon activity has been unclear. Therefore, we investigated it using a luciferase refolding assay. HSP90 refolds the structure and the activity of luciferase that is denatured by mild heating. In this assay, we detected luminescence produced by renatured luciferase to evaluate HSP90 chaperon activity. The level of luminescence was significantly reduced by treatment with a HSP90 inhibitor, geldanamycin (Figure 4). In contrast, matrine elevated the luminescence level significantly. These results suggest that matrine is an activator of HSP90 chaperon function.

### Extracellular HSP90 mediates matrine-induced axonal growth and SCI improvement

Beside its localization in the cytosol and on plasma membranes (Sidera et al., 2004), HSP90 is also secreted from neurons into the extracellular space (Schubert et al., 2009). Cytosolic HSP90, called “intracellular HSP90,” interacts with intracellular proteins (Tsutsumi and Neckers, 2007). In contrast, membrane-bound HSP90 and secreted HSP90 are called “extracellular HSP90,” and interact with proteins located at the plasma membrane and in the extracellular space (Tsutsumi and Neckers, 2007). Since client proteins of intracellular HSP90 and extracellular HSP90 are partially different (Tsutsumi and Neckers, 2007), the signal pathways of intracellular HSP90 and extracellular HSP90 might be different. Another group reported that treatment with recombinant HSP90 promoted neurite outgrowth in cultured central nervous system (CNS) neurons (Ishimoto et al., 1998). This report let us to hypothesize that matrine activates extracellular HSP90, leading to axonal growth and to improvement of SCI. To confirm this hypothesis, we investigated whether the specific blocking of extracellular HSP90 using HSP90 neutralizing antibody diminished matrine-induced axonal growth *in vitro* and improvement of SCI *in vivo*.

**Figure 5:**
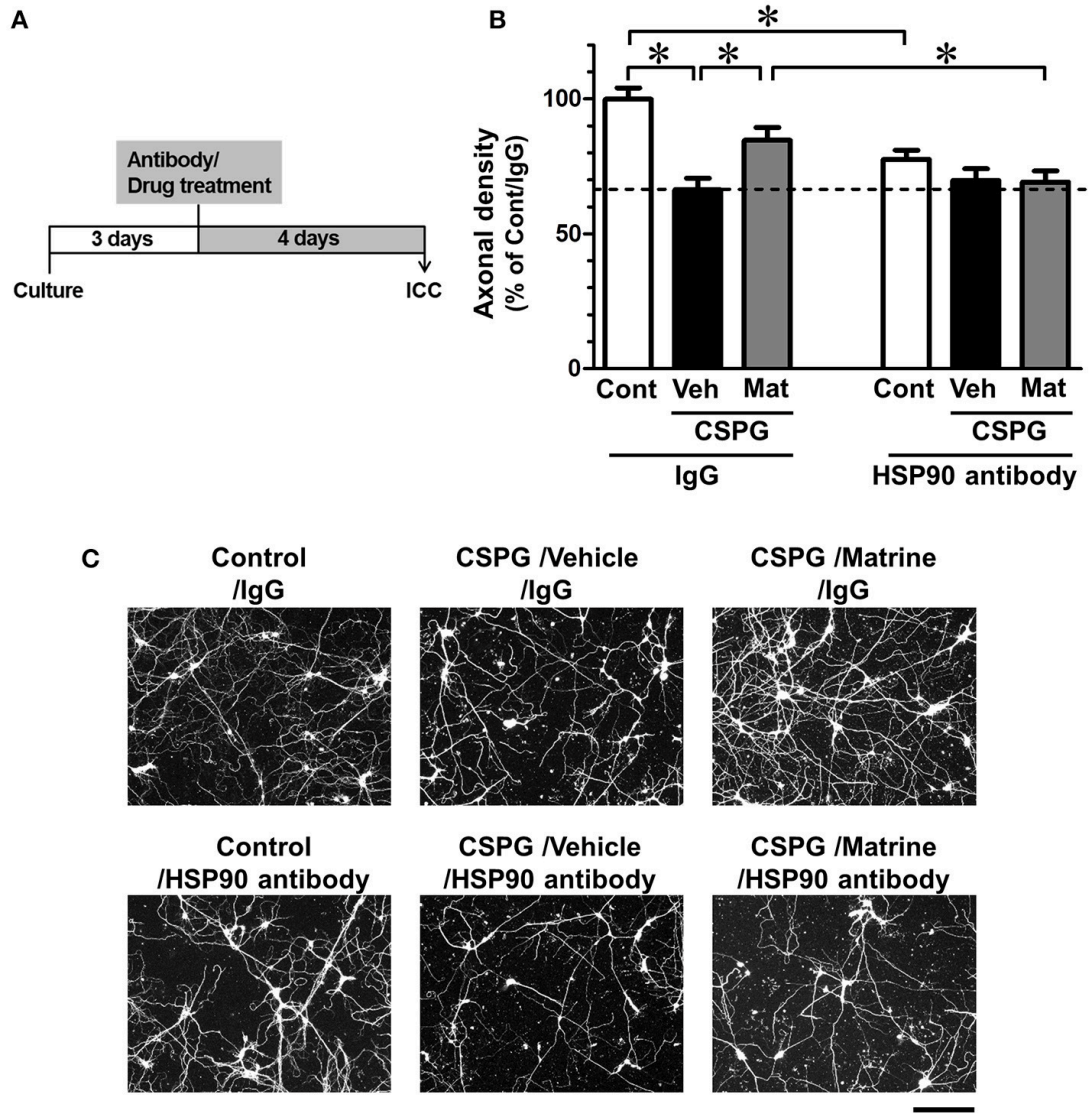
Specific inhibition of extracellular HSP90 diminishes matrine-induced axonal growth *in vitro*. **(A)** The time course of the experiment is shown. Mouse cortical neurons were cultured on CSPG-coated dishes for 3 days. The cells were treated with a normal mouse IgG or a HSP90 neutralizing antibody. Thirty minutes later, the matrine (10 μM) or vehicle solution was added. After additional incubation for 4 days, the neurons were fixed and immunostained for phosphorylated neurofilament-H (pNF-H) and microtubule-associated protein 2 (MAP2). **(B)** Density of pNF-H-positive axons per neuron was quantified. ^*^p < 0.05, one-way ANOVA followed by *post hoc* Dunnett's test. *n* = 7–13 captured images. **(C)** Representative images of pNF-H-positive axons are shown. Scale bar = 200 μm.

Cortical neurons were seeded on CSPG-coated dishes and treated with a HSP90 neutralizing antibody (1 ng/ml) or normal mouse IgG (1 ng/ml), followed by treatment with matrine (10 μM) or vehicle solution. The concentrations of the HSP90 antibody and the normal IgG were determined as the maximum dose at which the normal IgG had no effect on axonal growth (data not shown). Under treatment with normal IgG, axonal density was significantly decreased, and matrine significantly enhanced axonal growth on the CSPG coat (Figures 5B,C). In contrast, pre-treatment with HSP90 neutralizing antibody completely diminished the matrine-induced axonal growth on CSPG-coated dishes. Treatment with HSP90 antibody itself significantly decreased axonal density on PDL. In addition, we conformed that neurite outgrowth on PDL was reduced by FITC-geldanamycin, which is an impermeable HSP90 inhibitor and targets extracellular HSP90 (Supplementary Figure 4). Those findings indicate that the endogenous activity of extracellular HSP90 contributes to neurite outgrowth, and that matrine-induced axonal growth is mediated by extracellular HSP90 on CSPG.

**Figure 6:**
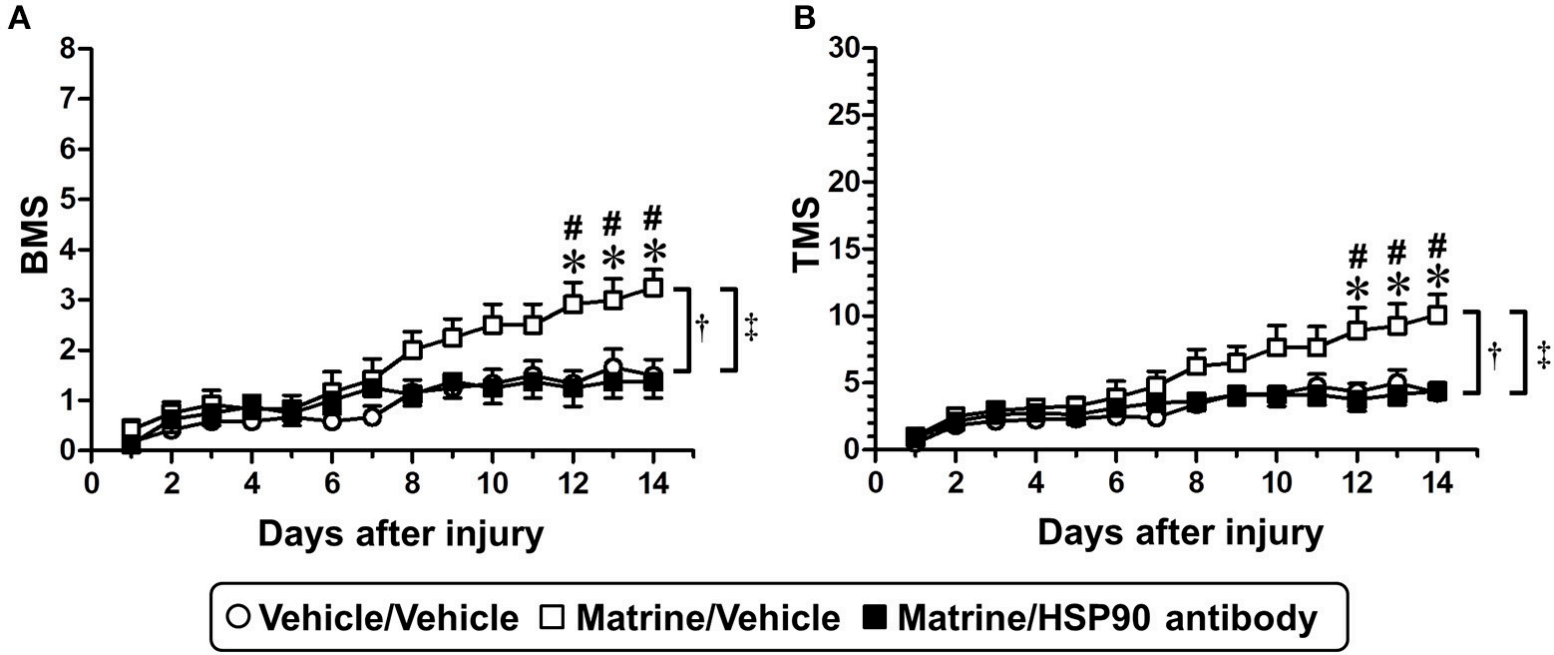
Specific inhibition of extracellular HSP90 diminishes matrine-induced functional recovery in SCI mice. SCI mice were continuously administered matrine (100 μmol kg^−1^) or vehicle solution (saline) orally. Matrine-administered mice were separated into two groups: one group was i.c.v.-injected with vehicle solution (artificial CSF), and the other one was i.c.v.-injected with HSP90 neutralizing antibody using an osmotic mini pump. BMS **(A)** and TMS (B) were measured to evaluate the motor function of the hindlimbs. **^†^***p* < 0.05, vehicle/vehicle vs. matrine/vehicle; **^‡^***p* < 0.05, matrine/vehicle vs. matrine/HSP90 antibody, drug x day interaction analyzed by two-way repeated measures ANOVA. ^*^p < 0.05, vehicle/vehicle vs. matrine/vehicle; #*p* < 0.05, matrine/vehicle vs. matrine/HSP90 antibody, *post hoc* Bonferroni tests. Vehicle/vehicle group: white circle, 6 mice, 12 hindlimbs, *n* = 12. Matrine/vehicle group: white square, 6 mice, 12 hindlimbs, *n* = 12. Matrine/HSP90 antibody group: black square, 4 mice, 8 hindlimbs, *n* = 8.

After SCI surgery, the HSP90 neutralizing antibody or vehicle solution (aCSF) was successively infused into the lateral ventricle using a micro-osmotic pump for 14 days. Matrine (100 μmol kg^−1^ day^−1^) or vehicle solution (saline) was administered orally for 14 days. In this experiment, SCI mice suffered trauma that was more severe than what is shown in Figure 1, to evaluate the therapeutic effect of matrine on severe injury. Vehicle/Vehicle-administered mice showed serious motor disfunction, indicated by a BMS score of 1.5 (Figure 6A) and a TMS score of 4.25 (Figure 6B) 14 days post injury. Fourteen days of matrine administration significantly enhanced the recovery of motor functions to scores of 3.25 on the BMS and 10.08 on the TMS. However, the matrine-induced functional recovery was completely diminished by the infusion of HSP90 neutralizing antibody (1.375 on the BMS and 4.375 on the TMS, 14 days post injury). The two-way repeated measures ANOVA yielded the following F and *p* values of the drug × day interaction among the groups: [Vehicle/Vehicle vs. Matrine/Vehicle] *F*_(13, 286)_ = 6.814, *p* < 0.0001 in BMS; *F*_(13, 286)_ = 5.855, *p* < 0.0001 in TMS; [Matrine/Vehicle vs. Matrine/HSP90 antibody] *F*_(13, 234)_ = 8.587, *p* < 0.0001 in BMS; *F*_(13, 234)_ = 6.352, *p* < 0.0001 in TMS; [Vehicle/Vehicle vs. Matrine/HSP90 antibody] *F*_(13, 234)_ = 1.453, *p* = 0.1365 in BMS; *F*_(13, 234)_ = 1.205, *p* = 0.2764 in TMS.

We investigated the effect of non-specific antibody on matrine-induced functional recovery in SCI mice (Supplementary Figure 5). All SCI mice were constitutively infused with normal mouse IgG to lateral ventricle using osmotic-mini pomp after SCI surgery. The mice were randomly separated to two groups and were administered with matrine (100 μmol) or vehicle (saline) for 28 days. BMS and TMS were measured to evaluate the motor function of hindlimbs. As a result, the motor function of matrine/IgG group was significantly improved compared with vehicle/IgG group. These results suggest that normal mouse IgG have no effect on matrine-induced functional recovery. Therefore, the reduction of matrine-induced functional recovery by HSP90 antibody was caused by specific inhibition of HSP90, not non-specific blocking. These results suggest that matrine-induced SCI improvement is also mediated by extracellular HSP90.

## Discussion

This study provided a new oral agent against SCI, matrine, which improved motor dysfunction in SCI mice (Figure 1) and increased the density of 5-HT positive axons inside the glial scar and within the caudal area (Figure 2). HSP90 is a direct target protein of matrine in neuron (Figure 3), and matrine enhanced HSP90 chaperon activity (Figure 4). Although binding of matrine to HSP90 was already suggested (Zeng et al., 2015), this study showed, for the first time, the pharmacological effect of matrine on HSP90 chaperon activity. Furthermore, we demonstrated that matrine-induced axonal growth and SCI amelioration were completely mediated by extracellular HSP90 (Figures 5, 6). Matrine is a novel kind of molecular-targeting drug that promotes functional recovery after SCI. Oral bioavailability of matrine is 17.1 ± 5.4 % in rats (Yang et al., 2010), and matrine passes through the blood brain barrier (Tang et al., 2013).

Our understanding of the functions of extracellular HSP90 in the CNS is still limited. Extracellular HSP90 regulates the migration of cerebellar granule cells (Sidera et al., 2004), and promotes the neurite outgrowth of cultured telencephalic and spinal neurons (Ishimoto et al., 1998). Exogenously applied HSP90 facilitates microglial activation, amyloid-β clearance, and the production of cytokines *in vitro* (Kakimura et al., 2002) and *in vivo* (Takata et al., 2003). The present study shows a novel finding regarding extracellular HSP90 in CNS, that is, that the activation of extracellular HSP90 may facilitate functional recovery after spinal cord injury. Few studies have shed light on HSP90 in SCI models so far. Proteomic studies of SCI models showed that the expression level of HSP90 around the lesion site was lower than that in the uninjured spinal cord (Zhou et al., 2014; Didangelos et al., 2016). Another study reported that nitrated HSP90, which has cytotoxicity, increased in the soma of spinal neurons after SCI, although its pathological contribution in SCI was unknown at the time (Franco et al., 2013). There are no reports showing the relationship between HSP90 and the functional deficit and/or improvement, and no reports focusing on extracellular HSP90 in SCI. Our study thus suggests, for the first time, the potential of extracellular HSP90 as a therapeutic target for SCI.

A variety of small compounds that interact with HSP90 have been identified, but almost all of them are functional inhibitors of HSP90 (Verma et al., 2016). The present study shows that matrine is the activator of the chaperon function of HSP90, and such a compound has not been shown yet. The folding function of HSP90 requires ATP binding and ATPase activity. Several HSP90 inhibitors, such as geldanamycin, inhibit HSP90 by preventing HSP90-ATP interaction (Pearl and Prodromou, 2006). Since matrine increased chaperone activity of HSP90 (Figure 4), matrine may increase ATPase activity of HSP90, leading to promoting the folding function.HSP90 plays roles in stabilization, activation, and/or renaturation of various client proteins via its chaperon activity, and thus affects the client protein-involved signal pathways (Taipale et al., 2010). Therefore, further investigations of matrine-induced HSP90 activation might lead to the clarification of novel pharmacological approaches to regulate signaling other than axonal growth.

A major response of drug binding in DARTS is stabilizing target proteins, which caused by masking protease recognition sites or by changing protein conformation to a resistant one (Lomenick et al., 2009). In our results, 1 and 10 μM doses of matrine and geldanamycin treatments prevented thermolysin-induced proteolysis (Figure 3C). In contrast, 100 μM matrine promoted the degradation of HSP90 (Figure 3C). Several reports showed that ligand binding made target proteins vulnerable against proteolysis (Pilch and Czech, 1980; Shigyo et al., 2015; Yang et al., 2017), which may be due to conformational alteration of the target proteins. Therefore, both reactions by matrine, HSP90 stabilization and digestion, may indicate the bound of matrine to HSP90.

Among the descending tracts, the raphespinal tract is one of the major tracts regulating locomotor function and is thought to be important for the recovery of motor function after SCI. Serotonergic axons act to modulate neuronal excitability in motor neurons (Perrier et al., 2013). Many reports about therapies for SCI showed a positive correlation between the growth of serotonergic fibers and the recovery of motor function (Teshigawara et al., 2013; Ruschel et al., 2015; Shigyo and Tohda, 2016). In the spinal cords of matrine-administered SCI mice, the density of 5HT-positive fibers increased inside the glial scar and within the caudal area (Figures 2A–C). Although the effect of matrine on the regulation of other tracts relating to motor function is unknown, the increase in 5-HT-positive fibers is possibly one of the critical phenomena contributing to the improvement of motor dysfunction induced by matrine.

The downstream signaling mechanism that promotes axonal growth following matrine-induced HSP90 activation is somewhat unclear. To clarify this mechanism, the identification of the clients mediating the signaling is essential. The clients of extracellular HSP90 are probably the proteins secreted into the extracellular space and/or binding to the cell surface: Extracellular HSP90 interacts with secreted matrix metalloproteinase 2 and changes matrix metalloproteinase 2 to an active form (Eustace et al., 2004), while extracellular HSP90 binds to human epidermal growth factor receptor-2 (HER-2), a membrane receptor, and assists HER-2-HER-3 heterodimer formation, which is necessary for heregulin-induced HER-2 activation and signaling (Sidera et al., 2008). Currently, we are comprehensively exploring client proteins of extracellular HSP90 in the fraction of the plasma membrane and cultured medium of neurons, and investigating the contribution of the proteins to matrine-induced axonal growth. It has also been reported, however, that extracellular HSP90 promotes cell migration by altering actin dynamics, particularly lamellipodia development and spreading in cancer cells (Sidera et al., 2008) and Schwann cells (Sidera et al., 2004). Cytoskeletal rearrangement is an important event during axonal growth, and CSPG inhibits the protrusive activity of lamellipodia as well as filopodia, resulting in dystrophic endballs (Tom et al., 2004). Therefore, actin rearrangement may be a later event during matrine-induced axonal growth. Clarification of the downstream signaling events following the activation of extracellular HSP90 by matrine treatment is our next goal.

In conclusion, this study shows that matrine is a new kind of drug, an activator of HSP90 chaperon function. Matrine improved motor disfunction in SCI mice via enhancing axonal growth, and the beneficial effect was completely mediated by extracellular HSP90, which is a novel target for SCI therapy. Our findings may lead to the development of new pharmacological approach by regulating HSP90 chaperon activity, especially molecular-targeting drug that activate extracellular HSP90 to promote functional recovery after SCI.

## Author contributions

NT, TK, and CT: designed the experiments and wrote the manuscript; NT: conducted the experiments and analyzed the data; CT: supervised all experiments and analysis.

## Declaration

The authors declare that this paper adheres to the principles for transparent reporting and scientific rigor of preclinical research recommended by funding agencies, publishers, and other organizations engaged with supporting research.

### Conflict of interest statement

The authors declare that the research was conducted in the absence of any commercial or financial relationships that could be construed as a potential conflict of interest.
