# Supplementary material for: Matrine Directly Activates Extracellular Heat Shock Protein 90, Resulting in Axonal Growth and Functional Recovery in Spinal Cord Injured-Mice
Source: Front Pharmacol. 2018 May 7;9:446. doi: 10.3389/fphar.2018.00446 (PMC5949560; doi:10.3389/fphar.2018.00446)
Supplement: Supplementary file 1 [file Data_Sheet_1.docx]

Supplementary Material

Matrine Directly Activates Extracellular Heat Shock Protein 90, Resulting in Axonal Growth and Functional Recovery in Spinal Cord Injured-mice

Norio Tanabe, Tomoharu Kuboyama, and Chihiro Tohda *

*** Correspondence:** Chihiro Tohda, Ph.D.: chihiro@inm.u-toyama.ac.jp.

# 1. Supplementary figure


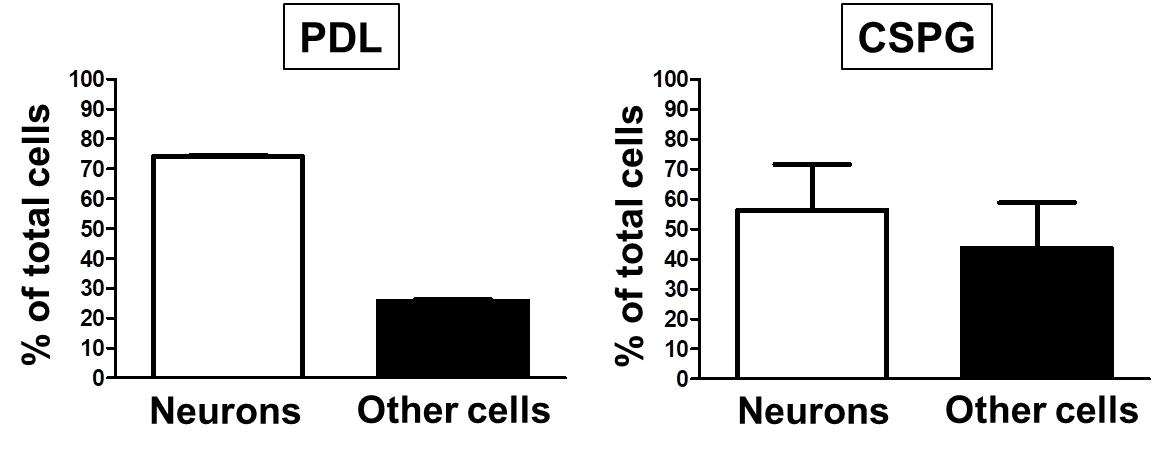


**Supplementary Figure 1 The purity of neurons in cortical neuronal culture.**

# Cortical cells were cultured for 7 days. The cells were fixed and immunostained for MAP2 (a neuron maker). DAPI was used for counterstaining. The number of MAP2-positeve neurons and DAPI were counted, and the purity of neurons was calculated. MAP2-negative cells were calculated as other cells.


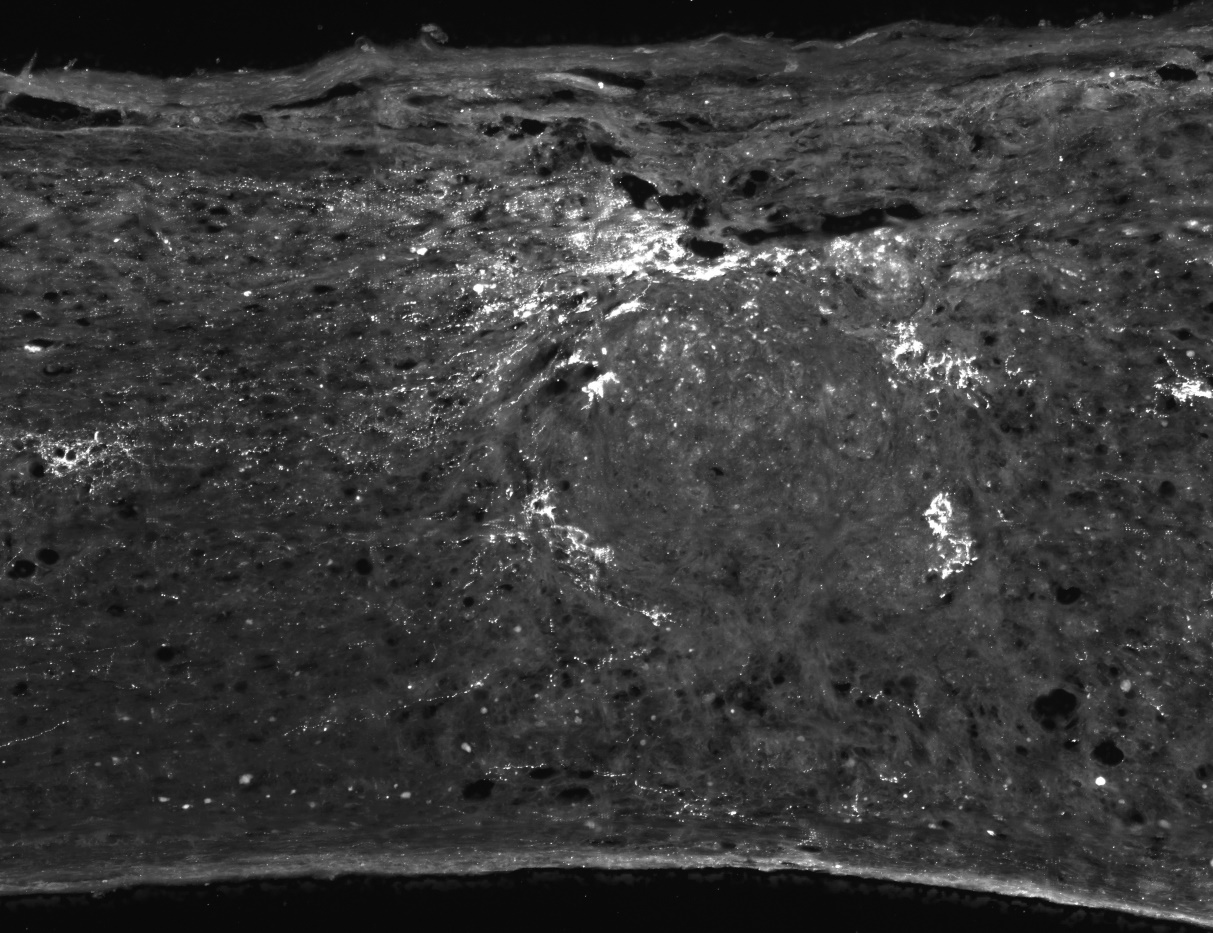


**B　Matrine : Lesion center**


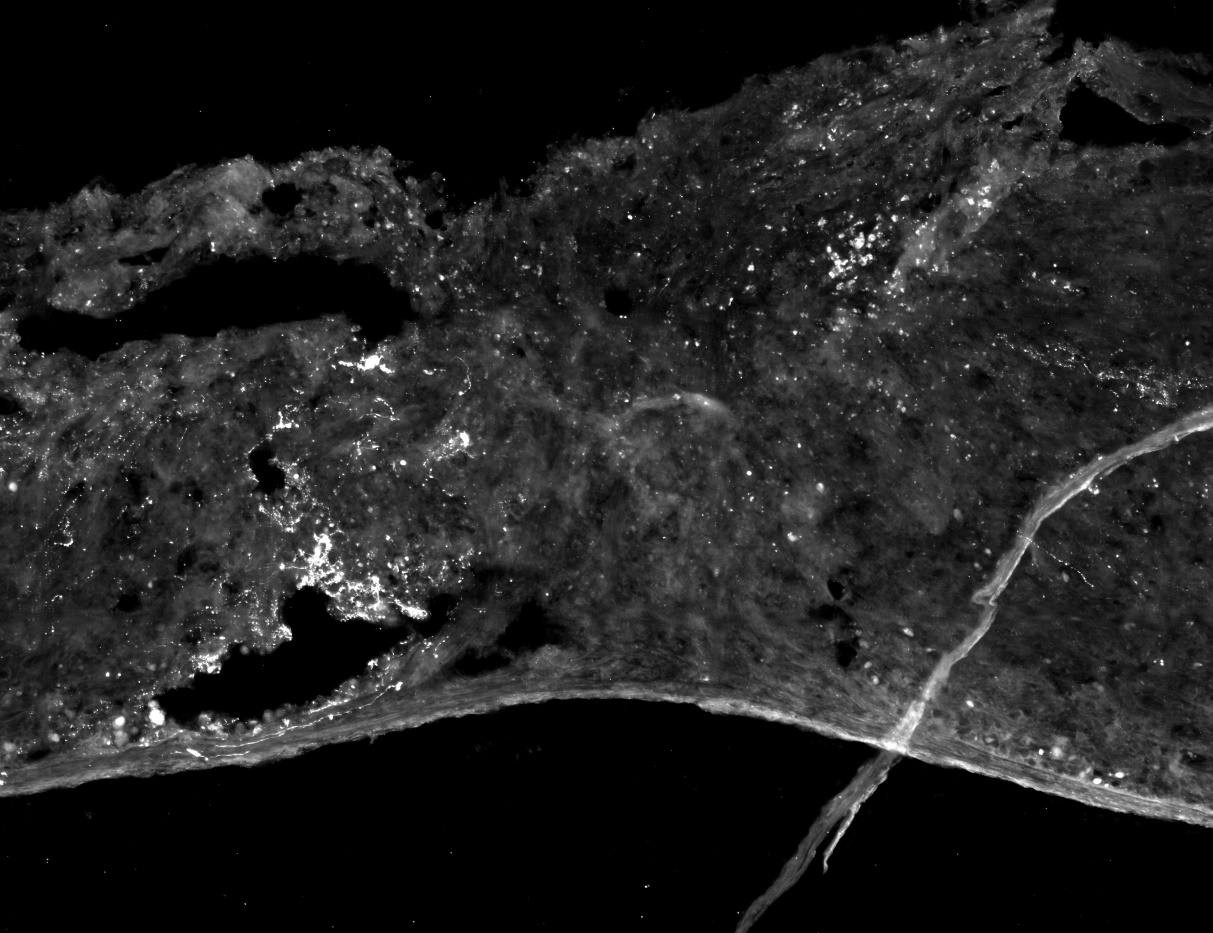


**Rostral**

**Caudal**

**Dorsal**

**Ventral**

**A　Vehicle : Lesion center**


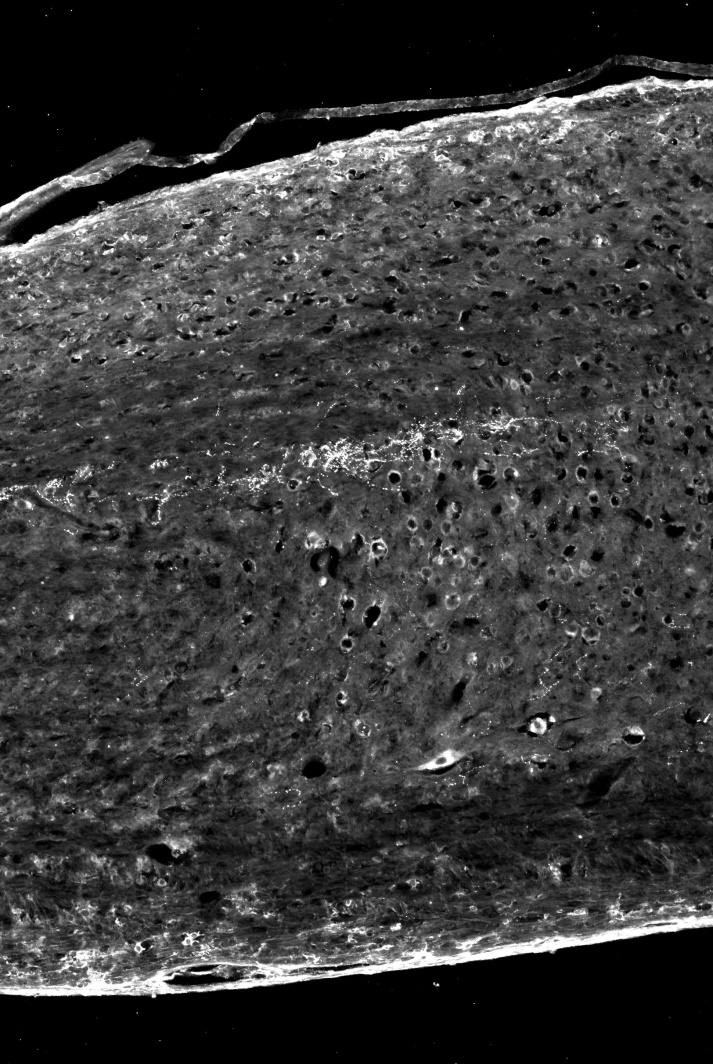

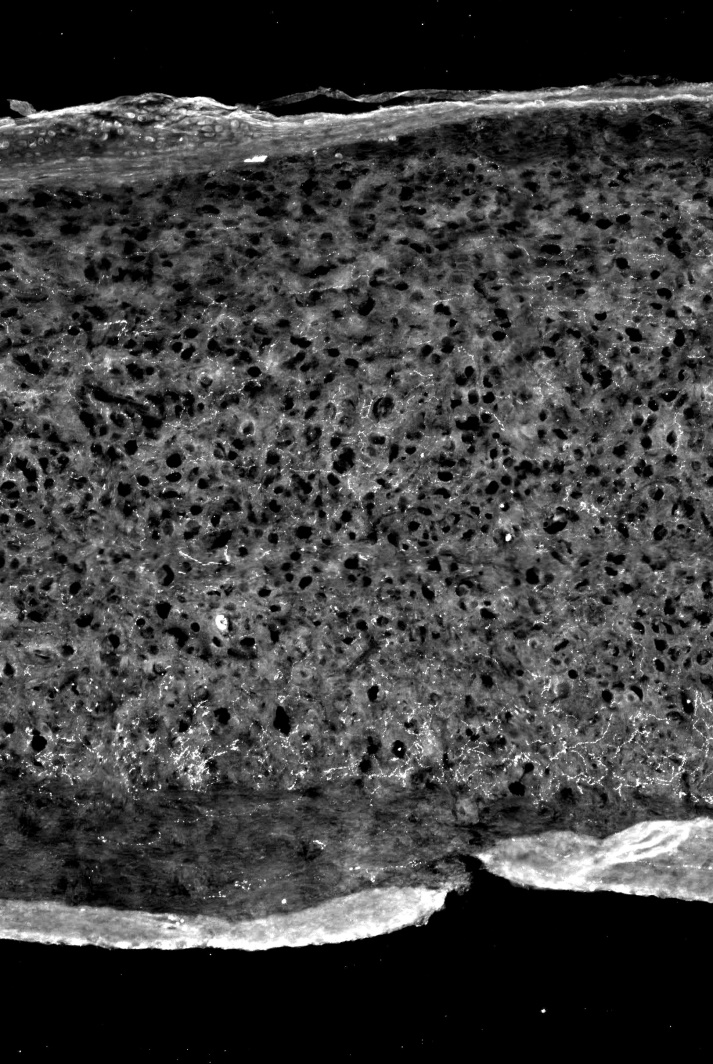


**C　Vehicle: Caudal site**

**D　Matrine: Caudal site**

**Rostral**

**Caudal**

**Dorsal**

**Ventral**

**Supplementary Figure 2 Representative images of spinal cord immunostained for 5-HT.**

At 31 days after injury, injured spinal cords were isolated from SCI mice that were treated with vehicle (A and C) or matrine (100 μmol kg^-1^; B and D). Sagittal sections of the spinal cords were immunostained for 5-HT, glial fibrillary acidic protein (GFAP). Representative images of immunohistochemistry for 5-HT are shown. (A, B) Whole images at the lesion center are shown. (C, D) Whole images at the caudal site are shown. The yellow dotted lines surround the glial scar. The red squares indicate the magnified areas showed in Figure 2A. The left direction of the images is rostral, and the upper side is dorsal. Scale bars = 500 μm.


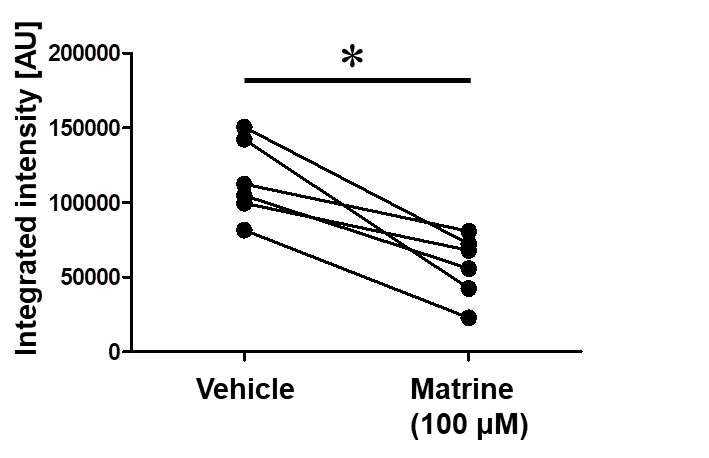


**Supplementary Figure 3 Matrine (100μM) promotes HSP90 digestion in DARTS.**

After DARTS reaction, a HSP90 was detected by western blot in the neuron lysates treated with matrine (100 μM) or vehicle. The integrated intensity of the bounds was quantified. * *p* < 0.05, paired *t*-test. n = 6 experiments.


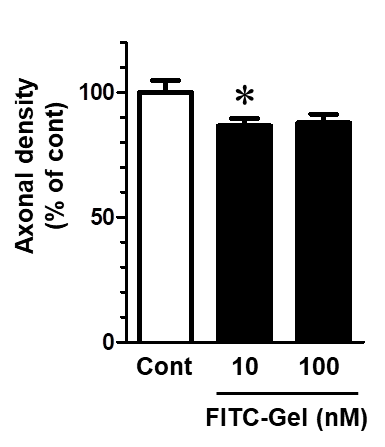


**Supplementary Figure 4 FITC-geldanamycin inhibits axonal growth.**

Mouse cortical neurons were cultured on PDL-coated dishes for 3 days. The cells were treated with FITC-geldanamycin (FITC-Gel; 10, 100 nM) or vehicle solution. After additional incubation for 4 days, the neurons were fixed and immunostained for phosphorylated neurofilament-H (pNF-H) and microtubule-associated protein 2 (MAP2). Density of pNF-H-positive axons per neuron was quantified. * *p* < 0.05, vs cont, one-way ANOVA followed by *post hoc* Dunnett’s test. n = 19-27 captured images.

**
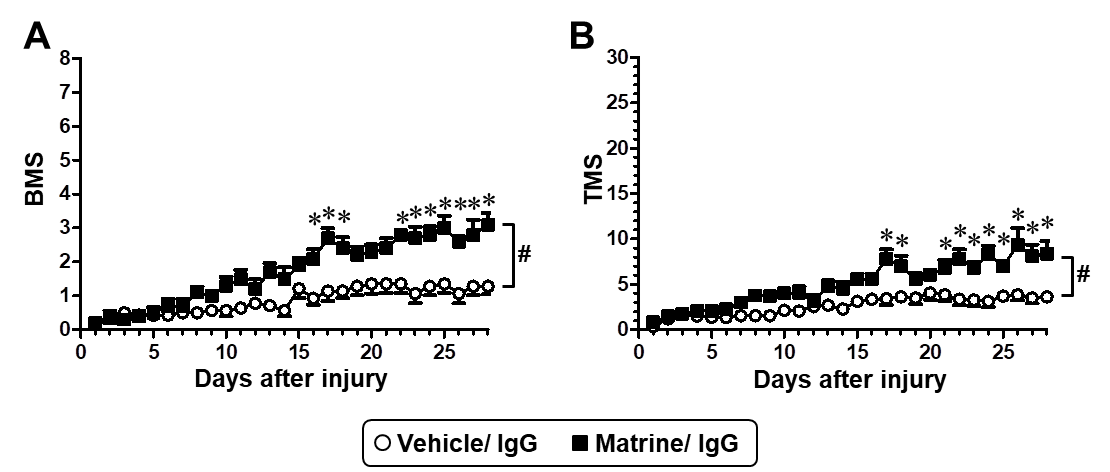
**

**Supplementary Figure 5 Non-specific antibody doesn’t diminish matrine-induced functional recovery in SCI mice.**

After SCI surgery, all SCI mice were constitutively infused with normal mouse IgG to lateral ventricle using osmotic-mini pomp. The mice were randomly separated to two groups and administered with matrine (100 μmol) or vehicle (saline) for 28 days. BMS (A) and TMS (B) were measured to evaluate the motor function of hindlimbs. # *p* < 0.05, vehicle/IgG vs matrine/IgG, drug × day interaction analyzed by two-way repeated measures ANOVA, F(27, 594) = 6.307 in BMS; F(27, 594) = 5.808 in TMS. * *p* < 0.05, vehicle/IgG vs matrine/IgG, *post hoc* Bonferroni tests. Vehicle/IgG group: white circle, 7 mice, 14 hindlimbs, n = 14. Matrine/IgG group: black square, 5 mice, 10 hindlimbs, n = 10.

# 2. Supplementary Video

**Supplementary Video 1.** The ambulation of a representative vehicle solution-treated SCI mouse at 31 days after SCI

**Supplementary Video 2.** The ambulation of a representative matrine-treated SCI mouse at 31 days after SCI
